# Denture Liners: A Systematic Review Relative to Adhesion and Mechanical Properties

**DOI:** 10.1155/2019/6913080

**Published:** 2019-03-03

**Authors:** Simone Kreve, Andréa C. Dos Reis

**Affiliations:** Department of Dental Materials and Prosthodontics, Ribeirão Preto Dental School, University of São Paulo (USP), Ribeirão Preto, SP, Brazil

## Abstract

**Purpose:**

The objective of this systematic review is to compare results concerning the properties of adhesion, roughness, and hardness of dental liners obtained in the last ten years.

**Methods:**

Searches on the databases LILACS, PubMed/Medline, Web of Science, and Cochrane Database of Systematic Reviews were supplemented with manual searches conducted between February and April of 2018. The inclusion criteria included experimental* in vitro* and* in vivo*, clinical, and laboratory studies on resilient and/or hard liners, assessment of hardness, roughness, and/or adhesion to the denture base, and physical/mechanical changes resulting from the disinfection process and changes in liners' composition or application.

**Results:**

A total of 406 articles were identified and, from those, 44 are discussed. Twenty-four studies examined the bond strength, 13 surface roughness, and 19 the hardness. Of these 44 studies, 12 evaluated more than one property. Different substances were used in the attempt to improve adhesion. Considering roughness and hardness, the benefits of sealants have been tested, and the changes resulting from antimicrobial agents' incorporation have been assessed.

**Conclusion:**

Adhesion to the prosthesis base is improved with surface treatments. Rough surfaces and changes in hardness compromise the material's serviceability.

## 1. Introduction

Liners have been widely used in dentistry to reshape prostheses surfaces in contact with soft tissues of the oral cavity [1]. Failure in adhesion, rough surfaces, and changes in hardness are favorable factors for microbial accumulation and compromise the liner's durability and the oral health condition such as denture stomatitis [2], implant loss [3, 4], peri-implantitis [4], and osseointegration delay, as well as respiratory problems [5] that can interfere with the rehabilitation treatment success and quality of life.

Liners are also used for prostheses fractures, remodeling of bone crests [6–8], and cleft palate [9], in cases of excessive resorption of the alveolus and occurrence of lesions on the mucosa [9, 10], and in tissue conditioning during implant healing [11], among others, acting to dissipate part of the impact of mastication [8, 12]. They are processed in laboratories (heat-polymerized) [13] and/or dentist offices (self-polymerized) because of their easy and quick application [14–17]. The term “soft liners” refers to a class of resilient materials used to reline denture base surfaces in contact with the occlusal stress-bearing oral mucosa [18].

Liners can be either hard [6, 16], usually made of polymethylmethacrylate [9, 10, 19, 20], or resilient [20–24], when plasticizers are added to the resin and the silicone elastomers [22, 24, 25]. Resilient liners are intended to be elastic, absorb energy, and act on the cushion effect [24]. Resilient reline materials are also classified as short- or long-term products. Long-term resilient denture liner materials maintain their resilience for more than 30 days and can be used for up to 1 year, while short-term liners are recommended for use for up to 30 days [26].

Liners are noninvasive and relatively more economical if compared to make a new denture [23, 27, 28]. Patients prefer resilient liners over the hard ones, because they improve comfort [14, 21, 28, 29].

However, they have some disadvantages, like presence of surface defects and porosity, residual taste after use, tendency to pick up odors [14, 30], water uptake [14, 24, 31], poor adhesion to acrylic resin [9, 31], proneness to change of color [7, 23], difficulty to clean [32], and premature hardening due to plasticizers' solubilization [10, 31].

A successful relining depends on the bond strength between the liner and the resin base [1, 6, 33, 34]. The lack of bonding leads to debonding, diminishing the procedure's longevity, and may occur due to an inefficient bond to the denture, or low cohesive strength [31]. According to Ahmad et al. [1], better adhesion is obtained when the materials' chemical properties are similar. Adhesion of liners to base polymers depends on the chemical composition of materials involved [19] and is influenced by the resin type, thermal cycle, and surface treatment [19, 31]. Excessive roughness results in microbial colonization and difficult hygiene. Liners are unstable in aqueous solutions; the hardness increases after water, saliva, and cleaning agents' absorption. Denture relining can be a factor of predisposition for prosthetic stomatitis.

The sealants' application [28, 29], surface treatments [35, 36], and physical-mechanical changes resulting from disinfection [17, 32, 37], among others, improve adhesiveness, reduce roughness, and maintain the liners' initial hardness.

Based on what has been presented, preserving the liners' physical-mechanical properties is a challenge. Considering its immediacy, simplified process, and economy, since the relining allows the use of the same prosthesis, it could be expected to grow demand especially more in dependent elderly care. This subject approach through a systematic review allows analyzing many studies' outcomes that have been carried out in attempt to improve these materials' limitations, such as debonding of denture base and changes in roughness and hardness that compromise its elasticity, assisting the clinicians in choosing the best product or technique. This systematic review covers studies published in the past 10 years aiming to assess the state of the art of liners, properties of adhesion, roughness, and hardness.

## 2. Materials and Methods

The question posed was as follows: Do the denture liners' modifications alter the adhesion, roughness, and hardness properties?

This systematic review was conducted according to the PRISMA (Preferred Reporting Items for Systematic Reviews and Meta-Analyses) report [38, 39] and registered on the PROSPERO database: CRD42018108821.

The review question, objectives of the study, eligibility criteria, and search and data analysis strategy were clearly stated in advance and incorporated in the protocol's content.

### 2.1. Defining Eligibility Criteria

#### 2.1.1. Search Methods

Studies reporting the properties of adhesion, roughness, and hardness of dental liners were identified by searching electronic databases and scanning reference lists of articles. Four databases were searched, LILACS, PubMed/Medline, Web of Science, and Cochrane Database of Systematic Reviews, using the following keywords: “denture liner” OR “reline” AND “soft liner” OR “surface roughness” OR “bond strength” OR “hardness” OR “hard liner.”

The literature survey was conducted from February to April of 2018 and included articles published between 2008 and 2018, in the Journal Citation Reports (JCR) indexed journals. This period was chosen for the review since the articles within that time interval depict the results of the main findings previously. Supplemental searches were conducted; the reference and citations' lists of the selected papers were reviewed in order to select potential inclusions.

#### 2.1.2. Types of Interventions

This systematic review was performed to answer the following questions: In patients wearing removable prostheses fitted with denture liners, does the bond strength of those materials alter? What has been used in the past 10 years to improve adhesion of denture liners to denture base? Do the modifications in the denture liners to improve the adhesion to the base of the prosthesis impair hardness and roughness values?

#### 2.1.3. Comparison

This study compares with the standard treatment, which in this case is applying the liner according to the manufacturer's instructions.

#### 2.1.4. Outcome Measures

The outcome measures were the effect of the intervention (denture liner) with some modification, as well as comparison between the effects of surface treatments with different substances on the properties of adhesion, roughness, and hardness. The main outcomes were defined when the article included in this review presented some adhesion, surface roughness, or/and hardness evaluation and showed a substantial result.

#### 2.1.5. Types of Studies

We selected and assessed papers published in English that met the inclusion criteria: experimental* in vitro *and* in vivo*, clinical, and laboratory studies on resilient and/or hard liners, assessment of hardness, roughness, and/or adhesion to the denture base, and physical/mechanical changes resulting from the disinfection process and changes in liners' composition or application.

Studies based exclusively on materials for denture base, unpublished data, critiques, case reports, and expert opinion papers should be excluded due to their high risk of bias [38]. Systematic reviews should also not be included.

#### 2.1.6. Study Selection

The study selection was carried out independently by two authors who adhered to the predefined eligibility criteria. Any disagreements between the two reviewers regarding the inclusion of studies were resolved by discussion.

#### 2.1.7. Assessment of Bias in Individual Studies

Risks were minimized by strictly following the keywords, the coherence of the selected abstracts, and analysis of articles published in selective editorial policy journals; this guarantees the quality of the individual studies.

Each of the included studies was then assessed for potential internal methodological bias such as the adequacy of randomization, incomplete outcome, and appropriate method of blinding.

## 3. Results

**Figure 1:**
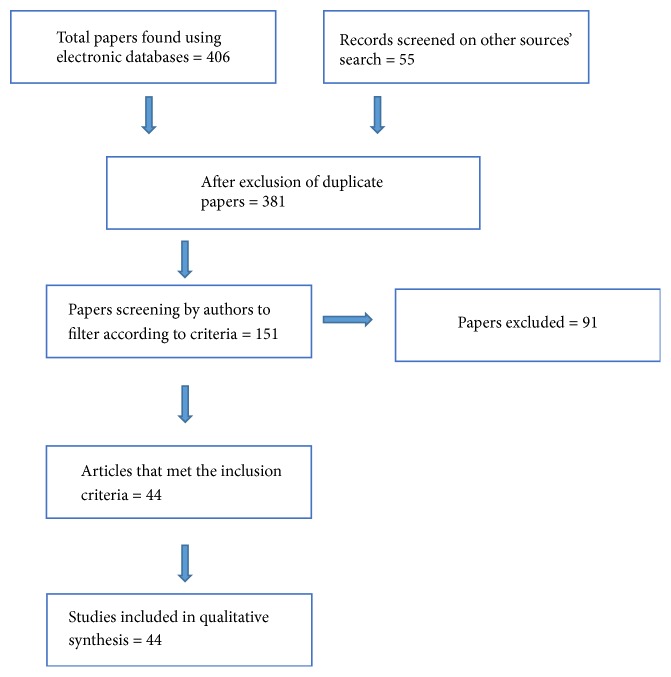
Flow of information through the different phases of the systematic review.

A total of 406 studies were identified on the initial screening. All abstracts were analyzed according to the PRISMA statement [38, 39]. Publications were identified as being relevant through the initial screening of titles and abstracts followed by screening of the full text. After exclusion of duplicates, 151 articles were selected for a complete assessment and, from these, 44 are discussed (Figure 1, Tables 1, 2, 3, and 4). Twenty-four studies examined the bond strength, 13 surface roughness, and 19 the hardness. Of these 44 studies, 12 evaluated more than one property. Most studies comprised* in vitro* evaluations, and only 3 were* in vivo* studies [25, 41, 42].

The articles were subdivided into categories since each article could address more than one property [8, 17, 19, 27, 29, 34, 35, 41, 43–45].

Considering the different commercial brands, Ufi Gel (VOCO) was the most commonly employed silicone-based liner, and Trusoft (BOSWORTH) was the most commonly employed resilient resin-base liner. Tokuyama Rebase II (Tokuyama) was the most used chairside hard liner.

Other materials such as Kooliner (GC America), Reline Soft (GC America), COE-SOFT (GC America), Sofreliner (Tokuyama), Mucopren Soft (Kettenbach), Elite Soft (Zhermack), and New Truliner (BOSWOTH) were also assessed often [1, 6, 12, 23, 25, 27, 37, 40, 41, 43, 46–48].

Types of intervention were as follows: comparison between the effects of surface treatments with different substances [12, 20, 35, 36, 49], bond tests between liners and different prosthetic materials [1, 10, 31], and assessment of the initial roughness of materials and that resulting from disinfection methods [17, 32, 44].

## 4. Discussion

Denture liners' materials have been widely used despite their substantial shortcomings. The use of solvents seems to improve the adhesion of the reliner to the PMMA base. Most cleansing agents compromise the hardness and elastic modulus. In addition, changes in roughness can lead to microbial colonization, increase the risk of oral and systemic infections, and decrease quality of life. Among the various disinfection methods, minor changes in the hardness and roughness properties were observed when incorporating antimicrobial agents into the liners.

### 4.1. Denture Liner Adhesion Mechanism

Aging [1] alters the adhesive properties of denture base polymers and liners [49] leading to flaws on the materials interface [41, 49, 50].

The bond between the prosthesis and liner begins with the dissolution of the resin by the solvent, swelling of surface layers, and evaporation of the solvent. The liner monomers diffuse, penetrate the resin pores, and form an interpenetrating polymeric network [51]. The larger the surface swelling, the deeper the porous layer and, as a consequence, the better the adhesion between the liner and denture base.

The bond strength between the liner and denture base was assessed [12, 15, 31, 49, 50] through primer application, where the layer of the GC resin primer was applied on the polyamide surfaces [52], through an adhesive such as a bonding agent that is a reline material partner [12], through sandblasting of the acrylic base resin surfaces with 50 *μ*m Al_2_O_3_ particles [20], through organic solvents, such as the application of an acetone solution and ethyl acetate solution [35], through application of a mixture of methyl formate and methyl acetate solution [51], and with changes in the prosthesis material like PMMA, preimpregnated with unidirectional glass fiber [12].

According to Ohkubo et al. [30], dentures used for an extended period of time are difficult to reline because microorganisms produce methyl mercaptan, which causes liner detachment even after the primer dissolution. Since bacteria penetrate to approximately 3 mm deep [30], more efficacy is obtained by reducing the base thickness and applying a high penetration primer, such as those based on dichloromethane.

#### 4.1.1. Silicone Liners

Silicone liners are mechanically superior and more durable than resin liners [35, 43]. However, they lack chemical adhesion [19, 31, 35], and adhesive flaws can be associated with the bonding agent [12]. Adhesive failures between the liner (silicone-type resilient denture liners) and prosthesis (heat-polymerized polymethylmethacrylate (PMMA)) increased from 13.8% to 60% after 30 days of storage in water [49], suggesting that their bonding gradually weakens over time.

Air abrasion with silica and silanization failed to improve bond strength of silicone resilient lining to the prosthesis (heat-cure acrylic), and the defects produced by the 30 *μ*m particles were not sufficient for the liner material penetration [20].

Organic solvents such as MMA (methylmethacrylate) and ethyl acetate improve silicone liners' adhesion to PMMA because they lead to softening and porosities that enhance adhesive penetration [33, 35]. Lassila et al. [12] found enhanced adhesion using ethyl acetate as bonding agent; Kim et al. [40] found better results using a primer or adhesive to adhere silicone liners to PMMA surfaces since they reduce the bubbles' formation during relining.

#### 4.1.2. Treatments to Improve Denture Liners' Adhesion to the Prosthesis

Treatment with acetic acid was comparable to that with tribochemical silica coating [52]. On the other hand, polymethylmethacrylate (PMMA) surfaces showed better adhesion with methyl formate-methyl acetate (MF-MA) than with resin liner bonding agents [51], composed of acetone and 2-HEMA, which is not volatile and obstructs the polymeric chains' interlocking, thus reducing bonding. There is no residual solution for MF-MA.

Another way to enhance the liners' adhesion to the prostheses is application of laser Er: YAG that alters prostheses surfaces, creating defects. Akin et al. [53] showed an increase in the silicone-based liners' bond strength to a UDMA base following laser application.

Considering experimental urethane acrylate oligomers-based photopolymerized soft liners, no significant difference in adhesion was observed after 1 day or 12 months of storage in water at 37°C [34]. This material seems to increase the liners' durability, which is usually of a few months.

#### 4.1.3. Liners' Adhesion to Different Types of Prostheses

To improve bonding between polyamide prostheses and self-polymerizable resin liners, the prosthesis treatment with tribochemical silica and 4-META/MMA-TBB (4-methacryloxyethyl trimellitate anhydride in methylmethacrylate initiated by tri-n-butyl borane) resin is recommended [36]. Polyamides are chemical resistant materials due to their high degree of crystallinity [33].

Ahmad et al. [1] found flaws in the liners' adhesion to a UDMA (photopolymerized urethane dimethacrylate) prosthesis due to its highly reticular nature that hinders the monomer penetration. In contrast, Akin et al. [53] found similar adhesion of the resilient liner to UDMA or PMMA prostheses. Adhesion of hard liners to thermoplastic acrylic resin was similar to that of conventional thermopolymerized acrylic resin; however, results were different for polyamide since these polymers are chemically resistant [33].

A weak adhesion between the resilient resin-base liner and prothesis was explained by the absence of monomers associated with nonreticulated amorphous polymers [50]. Nonetheless, glass fiber-reinforced PMMA showed increased adhesion to the liner since the fibers were previously filled with nonreticulated polymers containing PMMA islands in micrometric scale [12]. These exposed fibers were better dissolved by ethyl acetate.

#### 4.1.4. Antimicrobial Agents

It is important to assess changes in adhesion of prostheses and liners resulting from medicine incorporation. Antimicrobial additives can be a low-cost, effective alternative that does not require the patients' cooperation [12]. Pisani et al. [54] showed no changes in resin liner bonding considering immersion time or sodium perborate use, indicating that these do not affect the materials' dissolution. Alcântara et al. [55] showed that the addition of nystatin, miconazole, ketoconazole, or chlorhexidine diacetate in several dosages had no effect on the liner's adhesion to the prosthesis.

#### 4.1.5. Considerations Relative to Denture Liners' Adhesion

Poor adhesion creates a favorable environment to microorganisms and compromises the liner's durability. For silicone liners, the use of solvents seems to improve their adhesion to PMMA, since it favors the adhesive penetration and creates a mechanical blockage. For PMMA surfaces, the substitution of the most commonly found monomer (acetone and 2-HEMA) for a solution with better agent evaporation improves adhesion allowing the interlocking of the polymer chains.

### 4.2. Surface Roughness

There are several methods to remove contaminants from the liners, but it is important to assess their effects on the surface since cleaning solutions can penetrate the resin and change its morphology. In addition, immersion time and concentration can alter the polymer structure [32].

Self-polymerizable hard liners' roughness increases after immersion in sodium perborate and radiation with microwaves due to the immersion temperature and oxygen release by the perborate [17]. Bubbling from the oxygen release is a mechanical cleaning mechanism [17]. Izumida et al. [32] found a reduction in roughness associated with brushing and disinfection with sodium perborate and/or chlorhexidine gluconate and related it to cross-linked agents that reduce the acrylic resin solubility in organic solvents.

Brushing with only toothpaste and water increased roughness of silicone liner [32, 37], since toothpaste is composed of sodium carbonate, an abrasive agent.

No changes in roughness were found in one heat-polymerized denture base acrylic resin (Lucitone 550b) and another autopolymerized reline resin (Tokuyama Rebase Fast II) with different cleaning agents and this was associated with the short immersion time (1, 3, 21, 45, and 90 cycles of 10 seconds) [56]. Machado et al. [44] found an increase in roughness of the hard liner due to porosities formed from the release of residual monomers and plasticizers and from the increase in temperature during disinfection with microwaves. The increase in roughness was observed when organic solvents such as MMA were applied on PMMA as an attempt to improve adhesiveness to silicone-based liners [35], because these solvents degrade the surface and alter its morphology.

Values found for roughness of resin and silicone liners [22] exceeded the ideal clinical parameter (0.2 *μ*m) [57]. High values were also found by other authors [27, 44, 58]. Kutlu et al. [28] prepared the specimens on glass plates and obtained values above 0.2 *μ*m. Machado et al. [58] found initial roughness of 3.54 *μ*m in a resin-base liner. Methacrylate resilient liners are rougher than silicone liners due to their chemical structure, residual monomer content, polymerization method, monomers' volatility, and mixing technique [24, 43].

#### 4.2.1. Sealants' Application

Surface sealants protect liners against water absorption and damage from chemicals, saliva, food, and brushing and coating defects and reduce porosities and fissures [18, 29]. Their application reduced roughness produced by brushing in silicone and resin liners, with a more pronounced effect for siloxane-based material [18]. On the other hand, Kutlu et al. [28] showed no reduction in roughness when a sealant was applied to silicone-based and methacrylate-based liners. These findings are in agreement with another study [43]. Several situations increase liners' roughness, a favoring factor for bacteria accumulation. There is still no consensus on whether roughness is reduced when a surface sealant is applied.

### 4.3. Hardness

According to the specific ISO standards, liners can be categorized as type A (soft) or type B (extra soft) for measurements taken 24 hours after the preparation of specimens (ISO 10139-2:2009) [59].

A compilation associated with resilient liners comprises changes in hardness over time [42]. Hardness can be defined as penetration resistance [10], it increased in resin liners subjected to warm-water bath following polymerization, and it was associated with the reduction in residual monomers [6]. Mancuso et al. [60] also found an increase after aging that was associated with differences in type and content of plasticizers, leaching, and liquid absorption [17, 60]. Hardness of experimental photopolymerizable soft liners based on urethane acrylate oligomers was similar to that of silicone or acrylic resilient liners [34]. Conversely, Cazacu et al. [45] found higher hardness values for a thermostable silicone tested as liner, equivalent to that of addition silicone.

Chemical cleaning is the first choice to avoid liner damage. Immersion impacts malleability, ductility, and resistance to traction [19]. Immersion in different solutions increased the liners' hardness [17, 44, 46]. On the other hand, Rezende-Pinto et al. [27] found a reduction in self-polymerizable hard liners' hardness regardless of chemical solution or water immersion, before and after 30 cycles. Water diffuses through the resin until it saturates it and this results in surface softening.

Clinically, changes in hardness can also be caused by temperature fluctuations in the oral cavity and changes in pH [29], and, in the laboratory, they may still be affected by the type and concentration, immersion time, and composition of the cleaning solution. Changes in acrylic resilient liners occurred after 1 month of use by patients; and smoking patients showed higher hardness values, probably due to heat exposure. The frequent use of cleaners kept the liners soft and delayed their hardening process. Complete maxillary prostheses users presented higher values, associated with the materials' package. It is known that the pressure exerted by the denture during mastication accelerates the liner degradation. Complete monomaxillary prostheses exert greater occlusal strength than the bimaxillary prostheses. However, the authors showed no association between hardness and occlusal force after 1 month of the liner application. An increase in saliva acidity was associated with an increase in hardness, but this association cannot be generalized. Finally, use during sleep increased hardness, which was associated with individual and environmental factors [42].

Maintenance of materials' hardness is critical for their longevity; its effect, with and without sealants, varied among studies [18, 29]. Sealant application on resilient methacrylate can be effective in preserving hardness, since the solvent evaporates and creates a superficial layer resistant to degradation [29].

Given that soft liners' hardness is approximately 40 Shore hardness units (DIN 53505 and ASTM D2240/75), Santawisuk et al. [25] have enhanced the mechanical properties of an experimental silicone by adding synthetic silica. Comparing with silicone liners, it showed potential as a liner (Shore A hardness 41.3). Kasuga et al. [8] tested a fluorinated monomer of dodecafluoroheptyl methacrylate as soft liner material and observed Shore A hardness, similar to that of a commercially available silicone-based liner.

According to Izumida et al. [32], materials containing reticulation agents show greater stability in hardness when stored in aqueous solutions. Pisani et al. [48], on the other hand, found a hardness increase of both liners when stored in liquids. Hypochlorite was the solution that resulted in the greatest change.

Authors also failed to find significant changes when incorporating antimicrobial agents [61]. Chladek et al. [62] found no alterations in a silicone liner with the incorporation of silver nanoparticles in concentrations of up to 40 ppm. From 80 ppm, hardness and resistance to traction were considerably reduced.

It should be noted that the hardness has a direct relation with the viscoelastic properties which are responsible for distributing and absorbing the tensions generated during its clinical function [15, 19, 20]. The higher the hardness value, the lower the material's ability to absorb the impact of mastication [37]. Decrease in hardness values may lead to superficial changes and retention of oral pathogens. In addition, the silicone rubber-based soft lining materials enhance the growth of fungi such as* Candida albicans* on the presence of saliva [63, 64].

## 5. General Considerations

Failure of adhesion between the prosthesis and liner will compromise the procedure durability and favor microbial colonization. Adhesive failure may be associated with the bonding agent. The use of solvents in silicone-based liners seems to improve the adhesion of these to the PMMA base. A surface treatment is required to adhere liners to the polyamide denture base, either with acetic acid or with tribochemical silica. For PMMA surfaces, better adhesion is obtained with the same chemical properties of the liner and denture base. It is important to preserve the hardness values, so that the liner can maintain its elastic property.

Roughness surfaces and hardness changes favor microbial colonization and stomatitis. The selection of the liner should be based on the procedure's objective, considering serviceability, and expected results. The diversity of methods presented the properties in a diverse manner, showing that subsequent studies are necessary to meet better utilization and indication of liners regarding hardness, roughness, and adhesion. Based on the present results, further* in vivo* investigations with randomized controlled trials are necessary to compare the performance and properties of these denture liners' modifications in clinical use.

## Figures and Tables

**Table 1:** Included studies related to the bond strength.

Author/year	Method	Study objectives	Outcomes
Ahmad et al./2009	Shear bond strength	Bond strength, denture to reline materials	Higher bond strengths with similar compositions. PMMA (Meliodent), highest bond strength with (Meliodent RR), and UDMA (Eclipse) with Eclipse reline

Lassila et al./2010	Tensile bond strength	Bond strength, liners to fiber-reinforced and unreinforced PMMA	Sofreliner Tough, highest bond strength, and Eversoft, lowest

Więckiewicz et al./2014	Tensile and shear bond strength	Adhesion of silicone lining to denture base	A-Soft Line 30, the best adhesive properties

Mese and Guzel/2008	Tensile bond strength	Effect of storage on bond strength and hardness, resilient liners	Bond strength, lower as storage time increased. Greater changes, acrylic resilient liner

Atsü and Keskin/2013	Tensile bond strength	Bond strength, silicone denture liner, effects of surface treatment after thermocycling	Highest bond strength with adhesive. Surface treatment did not improve bond strength

Santawisuk et al./2013	Tensile bond strength	Comparing experimental silicone with lining materials	Silastic® MDX4-4210 silicone, greater mechanical properties

Ohkubo et al./2009	Shear bond strength	Bond strength immersing denture in methylmercaptan	Methylmercaptan causes liner detachment

Takahashi et al./2011	Tensile bond strength	Accelerated aging times on bond strength of soft liners	Mucopren Soft, higher tensile bond strength than Trusoft

Hamanaka et al./2016	Shear bond strength test	Bond strength of reline resin to injection-molded thermoplastic denture	Bond strengths' values varied. Bond improved, tribochemical silica coating and 4-META/MMA-TBB resin

Cavalcanti et al./2014	Tensile bond strength	Surface treatments on adhesion of silicone denture liners	Methylmethacrylate and ethyl acetate improved the adhesion of a silicone denture liner to PMMA

Kim et al./2014 [33]	Tensile bond strength	Bond strength, long-term soft denture lining	GC Reline Soft, highest bond strength. GC Reline Ultrasoft and Mucopren Soft, the lowest

Kanie et al./2009	Tensile bond strength and adhesive strength	Physical/mechanical properties, experimental light-curing soft lining	Tensile strength of UV-37, the lowest. No difference in adhesive strength between UV-35 and UV-37 at 1 day and 12 months

Dayrell et al./2012	Tensile bond strength	Bond strength and surface roughness of soft liners, sealer coating	Without surface sealer Mucopren Soft and Dentuflex, highest bond strength; Ufi Gel, intermediate; and Comfort Denso, the lowest

Takahashi et al./2009	Flexural strength test	Microwave postpolymerization (PP) on strength, acrylic resin intact and relined	New Truliner, smaller strength with PP microwave and effective only for Kooliner

Kim et al./2014 [40]	Tensile bond strength and transverse bond strength	Bond strength, relining resins, Acrytone, comparison, heat-polymerized acrylic and polyamide	Bond strength, reline resins and thermoplastic denture similar to acrylic resin. Polyamide, lowest

Maeda et al./2012	Peel bond strength	Bond of resilient denture liners to denture	All, adequate bond strength, used clinically for three years

Tanimoto et al./2009	Peel bond strength	Adhesive denture and denture liner	GC Reline Ultrasoft, lower adhesion

Koodaryan and Hafezeqoran/2016	Shear bond strength test	Bond strength, reline resin, polyamide and surface modification, acetic acid	Acetic acid, the greatest bond strength of MMA

Osathananda and Wiwatwarrapan/2014	Shear bond strength test	Bond strength, denture-reline resins, methyl formate-methyl acetate (MF-MA)	MF:MA ratio 25:75, enhances bond strength, denture and UNIFAST Trad, or Ufi Gel Hard

Akin et al./2013	Tensile bond strength	Bond strength, silicone denture liner, surface treatments	Denture base, silicone liner, similar bond strength. Lasing Eclipse resin, increased bond strength

Pisani et al./2009	Tensile bond strength	Sodium perborate, bond strength, degree infiltration, acrylic resin/denture liners	Kooliner, no difference in bond strength, immersion or sodium perborate. Mucopren Soft, highest tension, and Elite Soft the lowest

Alcântara et al./2012	Peel bond strength	Bond strength-antimicrobial soft liner, denture	Antimicrobial did not affect bond strength, resilient liner and denture

Chladek et al./2013	Tensile bond strength	Silver nanoparticle into denture liners	Tensile strength reduced. Bond strength of Ufi Gel, 40 ppm silver nanoparticle composites did not differ. Increase in bond strength, aging

**Table 2:** Included studies related to the hardness and roughness for soft denture liners.

Author/year	Method used	Study objectives	Outcomes
Kasuga et al./2011	Hardness values in Shore A durometer	Compare fluorinated monomer soft lining materials, conventional	No hardness difference, experimental fluorinated soft lining materials, Molloplast B

Mese and Guzel/2008	Hardness values in Shore A durometer	Storage duration on tensile bond strength and hardness, acrylic resin and silicone liners	Hardness, higher with increased duration of immersion

Santawisuket al./2013	Hardness values in Shore A durometer	Tensile strength, tear resistance, and hardness of experimental silicone elastomers (ESE)	Hardness, ESE increased with amount of silica filler (from 6 to 10 phr)

Kutlu et al./2016	Surface roughness tester	Sealer coating, roughness of soft lining	Roughness, methacrylate-based liners increased, denture cleanser. Sealer coating, no effect, roughness

Mante et al./2008	Hardness values in Shore A durometer	PermaSeal, hardness of soft reline	Sealer reduced saliva softening effect, methacrylate-based soft reline

Kim et al./2014 [33]	Hardness values in Shore A durometer	Hardness and bond strength of long-term soft denture lining	Hardness, 28-day increased compared to 24-hour

Kanie et al./2009	Hardness values in Shore A durometer	Evaluate experimental light-curing soft lining materials (ESLMs)	Hardness, UA-16, UV-32, and UV-35 similar to commercial denture liner

Badaró et al./2017	Hardness values in Shore A durometer and surface roughness tester	*R. communis *dentifrice (10%) on abrasiveness, hardness, and color change of a denture liner	Weight loss, roughness similar to Corega. Colgate, Corega Brite, roughness from 0.26 to 0.34 *μ*m. Brushing, no effect, hardness

Dayrell et al./2012	Surface roughness tester	Sealer coating, bond strength and roughness of liners	Palaseal coating, no effect, liners roughness. Without sealer coating, no difference observed on roughness

Machado et al./2012	Surface roughness tester	Roughness of denture resin, hard and resilient lining materials	No differences, initial roughness, Lucitone, Sofreliner, Tokuyama Rebase II, and New Truliner. Immersion 4%, chlorhexidine increased roughness. Ufi Gel Hard and Sofreliner, after 1 and 2 disinfection cycles

Cazacu et al./2009	Hardness values in Shore A durometer	Heat curable silicone, tested as a liner	Hardness, 59 ShA

Mainieri et al./2011	Surface roughness tester	Roughness, soft liners with and without surface sealer after brushing	Roughness, sealed COE-SOFT increased baseline, 1 month. Ufi-Gel with and without sealer coating, after 6 months

Machado et al./2011	Surface roughness tester	Roughness denture, hard and resilient lining materials	Roughness, Tokuyama Rebase II and Ufi Gel Hard similar or > New Truliner. Lucitone and Tokuyama Rebase II, no affected immersion desinfection

Mancuso et al./2012	Hardness values in Shore A durometer	Ageing effect, hardness, absorption, solubility, and color denture liners	Thermocycling influenced hardness

Leite et al./2010	Hardness values in Shore A durometer	Hardness values	Thermal cycling increased hardness, Elite Soft. Decrease for Kooliner

Pisani et al./2012	Hardness values in Shore A durometer and surface roughness tester	Color stability, hardness and roughness, denture liners cleansers' immersion	Hardness increased. Hypochlorite altered hardness. Elite Soft, highest roughness

Bertolini et al./2014	Hardness values in Shore A durometer	Hardness, chlorhexidine diacetate or chlorhexidine hydrochloride soft lining	Hardness, no changes, antimicrobial agents

Chladek et al./2013	Hardness values in Shore A durometer	Mechanical changes denture liners, silver nanoparticle	Hardness, greater 25 Sh. A, 10 ppm, 40 ppm

Urban et al./2014	Hardness values in Shore A durometer and surface roughness tester	Hardness and roughness, liners with antimicrobial	Softone roughness increased, miconazole and chlorhexidine. Trusoft did not increase. Hardness and roughness, little changes

**Table 3:** Included studies related to the hardness and roughness for hard denture liners.

Author/year	Method used	Study objectives	Outcomes
Urban et al./2009	Vickers hardness tester and surface roughness tester	Effect of water-bath postpolymerization (PP), degree of conversion, flexural strength, and microhardness, reline resins	Hardness increased by PP except Ufi Gel Hard

Machado et al./2009	Vickers hardness tester and surface roughness tester	Hardness and surface roughness, microwave and chemical disinfection, reline resins, denture resin	Hardness, Lucitone 550, not affected. Kooliner and DuraLiner II, increased, except Lucitone 550. Microwave 2 cycles, increased roughness. Tokuyama did not increase. Hardness, small decrease, 30 days

Izumida et al./2014	Surface roughness tester	Roughness, denture cleansers, reline resin	Roughness, reduction, brushing and sodium perborate and/or chlorhexidine gluconate

Machado et al./2012	Surface roughness tester	Roughness, denture, hard chairside and resilient lining materials	Initial roughness, no differences, Lucitone and Sofreliner, Tokuyama Rebase II and New Truliner. Chlorhexidine 4%, increased roughness, Ufi Gel Hard and Sofreliner, after disinfection

Dias Panariello et al./2015	Knoop hardness and surface roughness tester	Roughness (brushing, immersion). Hardness, color, Lucitone 550 (L), and reline resin	Roughness, decreased to L. Hardness, NaOCl and perborate, decreased to L. Hardness, decreased for T

Machado et al./2011	Surface roughness tester	Roughness denture, hard chairside and resilient lining materials	Roughness, Tokuyama Rebase II and Ufi Gel similar or < New Truliner. Roughness, Lucitone and Tokuyama Rebase II, not affected by immersion and disinfection

**Table 4:** Included studies related to *in vivo* studies.

Author/year	Observation period	Method used	Study objectives	Outcomes
Mutlay et al./2008	3, 6, and 12 months	Evaluation criteria: physical integrity, surface detail, adhesion, color, odor, plaque accumulation, resilience, hygiene, mucosal condition, and signs of fungal colonization	Clinical performance denture liners, 12 months	Roughening, posterior region

Bail et al./2014	Rats used palatal plates, 14 days	A roughness tester	Roughness, soft liners	Roughness, Dentuflex and Dentusoft, similar. Trusoft, rougher than Dentusoft. Ufi Gel P, lowest roughness (14-day)

Ogawa et al/2016	Original and 1-month hardness, after oral exposure	Shore D hardness	Denture liners changes, 1-month clinical setting	Hardness, changes influenced, patients' characteristics
